# Exploring the Magnetoelectric Coupling at the Composite Interfaces of FE/FM/FE Heterostructures

**DOI:** 10.1038/s41598-018-35648-1

**Published:** 2018-11-26

**Authors:** Dhiren K. Pradhan, Shalini Kumari, Rama K. Vasudevan, Evgheni Strelcov, Venkata S. Puli, Dillip K. Pradhan, Ashok Kumar, J. Marty Gregg, A. K. Pradhan, Sergei V. Kalinin, Ram S. Katiyar

**Affiliations:** 1 Department of Physics and Institute of Functional Nanomaterials, University of Puerto Rico, San Juan, 00936 PR USA; 2 Extreme Materials Initiative, Geophysical Laboratory, Carnegie Institution for Science, Washington, DC 20015 USA; 3 Department of Physics and Astronomy, West Virginia University, Morgantown, WV 26506 USA; 4 Center for Nanophase Materials Sciences, Oak Ridge National Laboratory, Oak Ridge, Tennessee 37831 USA; 5 Institute for Functional Imaging of Materials, Oak Ridge National Laboratory, Oak Ridge, Tennessee 37831 USA; 6 Institute for Research in Electronics and Applied Physics, University of Maryland, College Park, MD 207425 USA; 7 Smart Nanomaterials Solutions, Orlando, FL 32707 USA; 8 Department of Physics & Astronomy, National Institute of Technology, Rourkela, 769008 India; 9 National Physical Laboratory (CSIR), New Delhi, 110012 India; 10 Centre for Nanostructured Media, School of Maths and Physics, Queen’s University of Belfast, Belfast, BT7 1NN Northern Ireland UK; 11 Center for Materials Research, Norfolk State University, 700 Park Avenue, Norfolk, Virginia 23504 USA

## Abstract

Multiferroic materials have attracted considerable attention as possible candidates for a wide variety of future microelectronic and memory devices, although robust magnetoelectric (ME) coupling between electric and magnetic orders at room temperature still remains difficult to achieve. In order to obtain robust ME coupling at room temperature, we studied the Pb(Fe_0.5_Nb_0.5_)O_3_/Ni_0.65_Zn_0.35_Fe_2_O_4_/Pb(Fe_0.5_Nb_0.5_)O_3_ (PFN/NZFO/PFN) trilayer structure as a representative FE/FM/FE system. We report the ferroelectric, magnetic and ME properties of PFN/NZFO/PFN trilayer nanoscale heterostructure having dimensions 70/20/70 nm, at room temperature. The presence of only (00*l*) reflection of PFN and NZFO in the X-ray diffraction (XRD) patterns and electron diffraction patterns in Transmission Electron Microscopy (TEM) confirm the epitaxial growth of multilayer heterostructure. The distribution of the ferroelectric loop area in a wide area has been studied, suggesting that spatial variability of ferroelectric switching behavior is low, and film growth is of high quality. The ferroelectric and magnetic phase transitions of these heterostructures have been found at ~575 K and ~650 K, respectively which are well above room temperature. These nanostructures exhibit low loss tangent, large saturation polarization (P_s_ ~ 38 µC/cm^2^) and magnetization (M_s_ ~ 48 emu/cm^3^) with strong ME coupling at room temperature revealing them as potential candidates for nanoscale multifunctional and spintronics device applications.

## Introduction

Multiferroic magnetoelectric materials exhibiting simultaneous ferroelectric and ferromagnetic order parameters with the control and switching of the magnetic order parameter by electric field and vice-versa are the subject of cutting-edge research as they are promising for next generation nanoscale electronic and memory devices^1–5^. Due to mutual exclusiveness and frequent chemical incompatibility between magnetism and ferroelectricity, few single-phase multiferroic materials exist in nature, with notable examples such as BiFeO_3_, YMnO_3_, Pb(Fe_0.5_Nb_0.5_)O_3_, Pb(Fe_0.5_Ta_0.5_)O_3_, Pb(Fe_0.67_W_0.33_)O_3_, TbMnO_3_, etc^6–10^. The most well-studied is the lead-free single-phase multiferroic is BiFeO_3_ (BFO), having both ferroelectric (T_c_ = 1143 K) and antiferromagnetic (T_N_ = 643 K) phase transitions above room temperature. However, it is still not envisioned for practical device applications due to high leakage current and low ME coupling. Unfortunately the majority of the other single-phase multiferroic materials are only multiferroic at cryogenic temperatures (magnetic/ferroelectric transitions are below room temperature). The ME coupling is also observed to be weak due to large independency of the magnetic and ferroelectric ordering in single-phase multiferroics^2,8,9^. An alternative route towards the goal of strong room-temperature ME coupling is to explore heterostructures composed of alternatively ferroelectric and ferromagnetic layers, or through development of composites^8–10^. The development of sophisticated thin film growth techniques has allowed the fabrication of ultra-high purity atomic-scale thin films free from extended defects, allowing a platform for the exploration of ME coupling in epitaxial heterostructures. Moreover, researchers have proceeded to design and develop artificial composite nanostructures to achieve large polarization and magnetization with strong ME coupling above room temperature^1,7–9^. The obvious choice for a high figure of merit ME nanostructure is a multilayer structure with an atomically smooth interface between the magnetic and ferroelectric layers. Magnetic materials with high magnetostriction, high Curie temperature (T_c_), and large magnetization, as well as ferroelectric materials with large polarization, dielectric constant and piezoelectric coefficients, are required to produce strong ME coupling in the heterostructures^8,9,11^. Achieving a large ME coupling in thin films with important implications for device applications is a long-standing scientific challenge^2,7–9^. In heterostructures, the spinel ferrites are widely adopted as the magnetic candidates due to their good thermal, chemical, and structural stabilities along with high resistivity^9,12^. The ME coupling may arise due to coupling between electrical and magnetic order parameters or due to cross coupling between magneto- and electro-striction and magnetic order parameters or indirectly via lattice strain and interfacial electronic reconstruction (charge coupling)^2,8,9^. For composites, the ME effect is a product tensor property of the piezoelectric and piezomagnetic coefficient that results from the cross interaction between the two phases^9–11^.

Several theoretical and experimental investigations have been reported that achieve large ME coupling for various composite bulk and thin film structures^11,13–22^. Strong ME coupling has been observed in different layered composite structures such as: trilayers of BaTiO_3_/BiFeO_3_/BaTiO_3_, bilayers of PbZr_0.57_Ti_0.43_O_3_/NiFe_2_O_4_, bilayers and superlattices of Ba_0.7_Sr_0.3_TiO_3_/La_0.7_Sr_0.3_MnO_3_, PbZr_0.2_Ti_0.8_O_3_/La_0.8_Sr_0.2_MnO_3_ heterostructures, PbZr_0.57_Ti_0.43_O_3_/CoFe_2_O_4_ bilayer and multilayers and BaTiO_3_/CoFe_2_O_4_ multilayer heterostructures^8,9,13,23,24^. The route to understanding the ME coupling has typically proceeded via first-principle approaches which have yielded a wealth of essential information on the complex interplay of spin-dependent interfacial charge transfer, chemical bonding, and electrostatic screening in these systems^17,18^. Theoretical calculations of the strain-mediated ME coupling necessitate the determination of three factors: (i) the deformations induced in the magnetic phase by the applied magnetic field; (ii) the degree of strain transmission through the interface between the two ferroic constituents of the hybrid system; and (iii) the response of polarization and dielectric susceptibility of the ferroelectric phase to changes in the lattice strain. In epitaxial heterostructures, the mechanical coupling between the two phases is very strong due to possible perfect mechanical strain transmission^17–21,25^.

The Pb(Fe_0.5_Nb_0.5_)O_3_ (PFN)/Ni_0.65_Zn_0.35_Fe_2_O_4_ (NZFO)/Pb(Fe_0.5_Nb_0.5_)O_3_ (PFN) trilayer heterostructure has been chosen for the present study to observe enhanced ferroelectric and magnetic properties with strong ME coupling above room temperature. PFN is a well-known multiferroic material with a ferroelectric T_c_ between 379–385 K having high dielectric constant (~3000 at RT), low loss tangent (0.01) and high piezoelectric coefficient (d_33_ ~ 145 pC/N); it also shows strong ME coupling near its T_N_ ~ 150 K^26–29^. Recently the existence of ferroelectric, ferromagnetic, and ferroelastic properties in PFN with interesting ferroelectric and magnetic properties have been reported^30^. Nickel zinc ferrites (NZFO) are soft magnetic materials with high saturation magnetization, low coercivity, high resistivity, reasonable magnetostriction, low dielectric losses, high dielectric constant and high magnetic Curie temperatures. Ni_0.65_Zn_0.35_Fe_2_O_4_ has been chosen for the present work as this composition exhibits the combination of highest saturation magnetization (86 emu/g), high resistivity (~2 × 10^6^ Ω cm) and high magnetostriction (10^6^ λ_100_ = −34) in the entire Ni−Zn series with a magnetic T_c_ of ~663 K^15,17,31–33^.

Here we have investigated the ferroelectric and magnetic phase transition, tip induced polarization switching and ME coupling properties of PFN/NZFO/PFN at room temperature. We find that these nanostructures show saturated large polarization, soft magnetization with both ferroelectric and magnetic phase transition well above room temperature. The observation of strong ME coupling at room temperature make this nanostructure a potential candidate for multifunctional and spintronics nanoscale device applications.

## Results and Discussion

### Structural and Morphological Characterization

**Figure 1:**
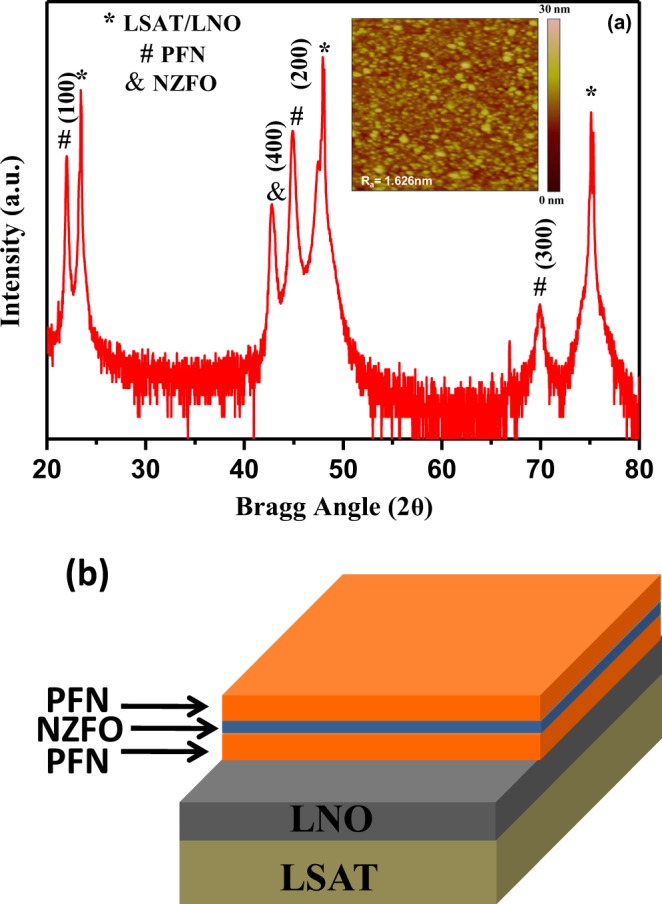
(**a**) XRD pattern and atomic force micrograph (inset) of PFN/NZFO/PFN heterostructures at room temperature. (**b**) The schematic diagram of PFN/NZFO/PFN heterostructures.

In order to obtain knowledge on the phase purity, crystalline quality, interface and structural information in detail, the films were investigated utilizing high-resolution XRD. Figure 1(a) shows the XRD patterns of PFN/NZFO/PFN trilayer heterostructure grown on LaNiO_3_ (LNO) buffered (LaAlO_3_)_0.3_ (Sr_2_AlTaO_6_)_0.7_ (LSAT) substrate recorded at room temperature. The θ−2θ large angle X - ray scans (20° to 80°) showed only diffraction peaks from the substrate and (00*l*) pseudocubic reflections from the heterostructure confirming that these films are highly oriented in nature. The peaks with symbols *, # and & correspond to the diffraction peaks of LSAT/LNO (overlapped), PFN and NZFO, respectively. The epitaxial character and the confirmation of the layered nature within the heterostructures were also confirmed by TEM studies (see below). We did not observe any extra reflection peaks that would be indicative of secondary phases and peaks from lead-deficient pyrochlore phases suggesting the high purity of the grown films. All of the observed peaks were assigned to PFN and NZFO, corresponding to perovskite phase with the monoclinic and cubic phase crystal structure respectively^31,34^. In order to explore the effect of strain on the ferroelectric, magnetic and magnetoelectric properties of this heterostructure, the effective misfit strain was calculated^35^. Note that the LSAT substrate has a cubic crystal structure with the lattice parameter a = 3.868 Å, whereas bulk LNO has a rhombohedral crystal structure with the lattice parameter a = 3.838 Å. The LNO electrode has been used in our case as an oxide electrode which is closely lattice-matched with substrate. It was calculated that LNO on LSAT experiences a small in-plane tensile strain of 0.78%. The peak positions of PFN and NZFO in the heterostructures have been compared to the stress free bulk values of PFN and NZFO. The individual peaks of the heterostructures are found to be shifted towards lower angle compared to the stress free bulk samples. The effective misfit strain experiencing by PFN and NZFO layers are found to be −1.02% and −0.91% respectively.

The inset of Fig. 1(a) shows the surface morphology of the top layer of PFN/NZFO/PFN nanostructure grown on LNO/LSAT substrate. The surface topography captured by AFM in the contact mode on a scan size of 5 × 5 *μ*m^2^, lacks granular structure, with average surface roughness (*R*_a_) and root mean squared roughness (*R*_*rms*_) being ~1.62 and 2.05 nm respectively. It is clearly seen that the upper surface of heterostructure is smooth and very homogeneous, free of microcracks, pores or holes. The smooth surface with low roughness is required for improvement of functional properties. Figure 1(b) depicts a schematic diagram of the PFN/NZFO/PFN heterostructures.

### Transmission Electron Microscopy Characterization

**Figure 2:**
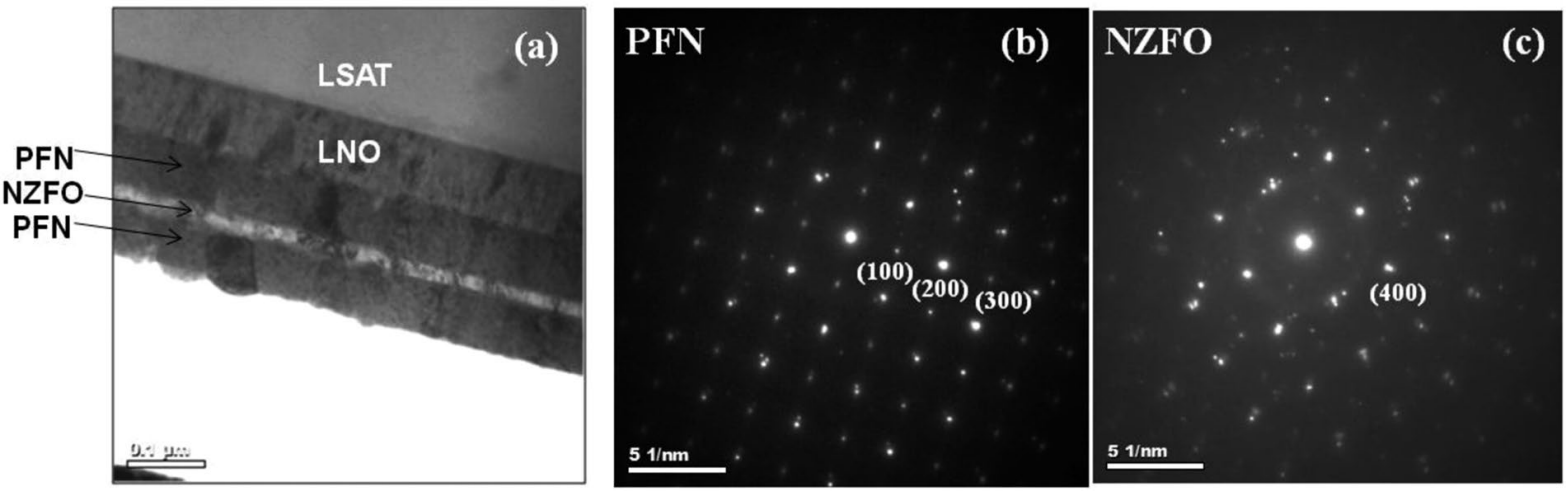
(**a**) Cross-sectional TEM image, SAED patterns of (**b**) PFN and (**c**) NZFO of the PFN/NZFO/PFN heterostructures.

The multiple interfaces that exist in the heterostructures can lead to carrier confinement effects, magnetic interactions, and lattice strain effects at the interface that play an important role in determining the physical and functional properties of the material^36–39^. Therefore, detailed studies of the interface structure are essential to understand many novel physical phenomena observed in heterostructures. Energy dispersive X-ray (EDX) in conjunction with transmission electron microscopy (TEM) was used to study the crystal structure, strain distribution, and local chemical composition of the heterostructure respectively. Figure 2(a) shows the cross-sectional TEM image of the (00*l*) - oriented PFN/NZFO/PFN heterostructures grown on LNO buffered LSAT substrate. The heterostructure exhibits one NZFO layer of thickness ~20 nm sandwiched between two PFN layers of thickness 70 nm, with total film thickness of ~160 nm. The layers in this heterostructures are distinctly separated from each other; darker bands correspond to the PFN layers and the brighter one to the NZFO layer having sharp and well-defined interfaces as seen from the TEM image. The distinct contrasts of the PFN image represent the domains separated by domain walls. Selected-area electron diffraction (SAED) patterns were collected corresponding to each layer and confirms the epitaxial growth of the layers in this heterostructure. The SAED patterns shown in Fig. 2(b,c) mainly represent the diffraction patterns from PFN and NZFO layer, respectively. TEM images show that the growth direction of PFN and NZFO layers is (*l*00), as seen in the XRD patterns. The measured lattice spacing from SAED pattern for PFN is 4.065 Å corresponding to the (100) planes whereas measured lattice spacing for NZFO is 2.20 Å corresponding to the (400) planes which are in agreement with the existing literature. Scanning transmission electron microscopy - Energy dispersive X-ray (STEM-EDX) were also used to analyse the existence of all individual elements (chemical composition) with their required stoichiometry of the PFN and NZFO layers in the heterostructure.

### Tip Induced Polarization Switching Studies

**Figure 3:**
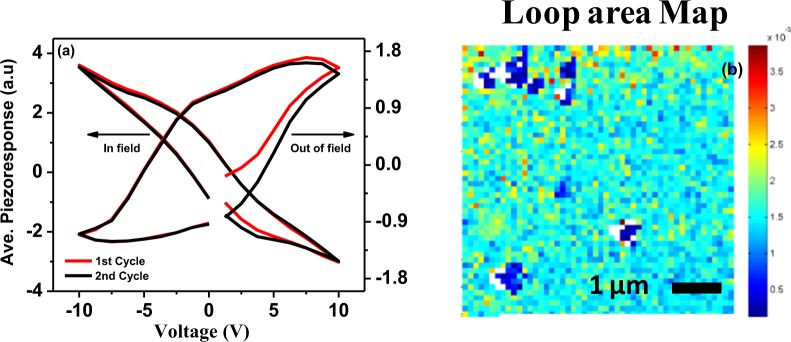
Band excitation PFM (**a**) out of field and in field piezo response hysteresis loop averaged over 50 × 50 grid of points (**b**) loop area map showing spatial distribution of switching behavior of PFN/NZFO/PFN heterostructures at room temperature.

Our previous investigations have shown evidence of the ferroelectric behaviour of PFN thin films, most noticeably via the dramatic collapse in the hysteresis loop upon reaching the Curie temperature^40^. Of more interest in this case is to probe the local variability of ferroelectric properties, which may be important to understanding the device properties and potential routes towards optimization of the field-induced strain response, Band Excitation PFM (BE-PFM) measurements have been carried out as shown in Fig. 3((a,b)). PFM measures the dynamic electromechanical response of the sample at the nanoscale when an ac excitation and a dc voltage is applied to the conductive AFM tip which is in contact with the surface^41,42^. It may be noted that PFM is only measuring the top layer of the heterostructure, with resolution typically ~20 nm (the contact radius of the tip) for each grid on the sample. The piezoresponse was measured as a function of tip voltage in the in field (V_DC_ on) and out of field (right after switching V_DC_ off), regimes as shown in Fig. 3(a). The out of field piezoresponse loop is fitted with phenomenological function and the value of R_+_, R_−_, V_+_, V_−_ and work of switching for cycle 2 are found to be 0.095 × 10^−3^, −0.744 × 10^−4^, 5.16 V, −4.933 V and 0.00171 respectively. Where R_+_, R_−_ represent the remanent polarizations and V_+_, V_−_ represent coercive bias. The on-field response generally includes stronger electrostatic effects, and is noticeable via the change in loop orientation. The off-field response resembles that measured for our pure PFN thin film reported earlier, and shows minimal variations by cycle. As PFM spectroscopy gives the advantage of functional mapping^41,42^ of the sample at the nanoscale, the distribution of ferroelectric loops (ave. piezoresponse vs voltage) of a loop area map of a 5 *μ*m × 5 *μ*m area of the sample overlaid with 50 × 50 grids is shown in Fig. 3(b). The distribution of the ferroelectric loop area is almost uniform in a wide area, suggesting that spatial variability of ferroelectric switching behavior is low, and film growth is of high quality. Note that the white pixels observed in this map are the locations where the fitting was poor due to low signal-to-noise ratio (i.e. these are non-ferroelectric defects on the surface of the thin films). Note also, the presence of ferroelectricity is further confirmed by bulk P-E loop measurements which will be discussed in the following section.

### Dielectric and Ferroelectric Properties

**Figure 4:**
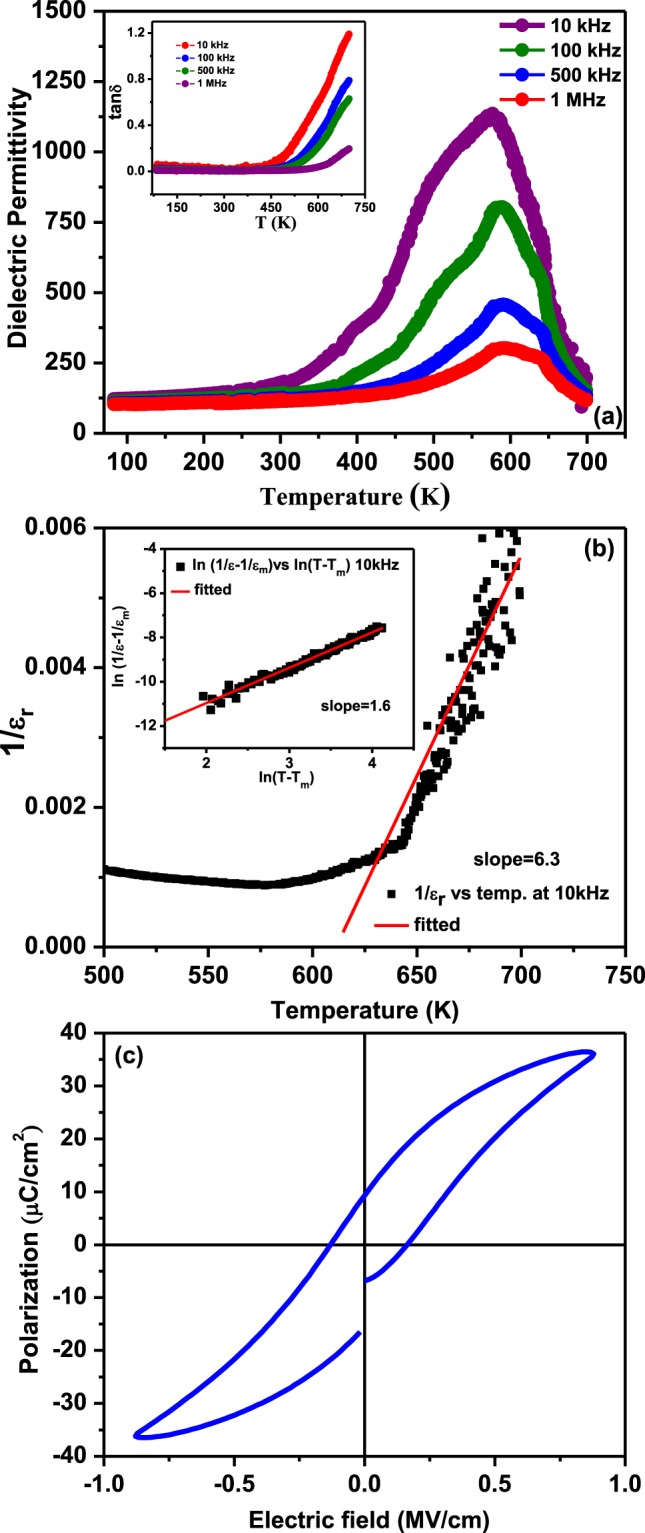
(**a**) Temperature dependence of relative dielectric permittivity and loss tangent (inset) at different frequencies (**b**) Curie Weiss behavior (**c**) Ferroelectric (P–E) hysteresis loops of PFN/NZFO/PFN heterostructures at room temperature.

In order to probe the ferroelectric phase transition, investigations into the temperature dependence of dielectric properties have been carried out. Figure 4(a) shows the variation of dielectric constant and loss tangent (inset) of the heterostructure across a wide range of temperature (80–700 K) at different frequencies. The dielectric constant increases slowly with increasing temperature up to 300 K and then increases rapidly with increase of temperature up to its maximum value and then decreases. The ferroelectric to paraelectric phase transition temperature (T_c_) in our case is found to be 575 (+/− 5) K for 10 kHz and it increases towards higher temperature with increasing frequency which might be due to charge trapping at the interface due to the termination of the ferroelectric polarization or Maxwell-Wagner space charge across the trilayer heterostructure. It may also be noted that pure PFN itself shows a diffuse type of phase transition behavior in bulk, single crystal and in thin film form, so the interface with NZFO is not the only one that can be responsible for this diffuse phase transition behavior^26–30^. A modified Curie-Weiss law has been utilized to test the diffusivity of phase transition;

1 $$\frac{1}{\varepsilon }-\frac{1}{{\varepsilon }_{max}}=\frac{{(T-Tm)}^{\gamma }}{c}$$

Here c is a constant and γ is the critical exponent which is the measure of diffuseness. The value of γ varies from 1 to 2. For sharp phase transition (ideal ferroelectrics) γ = 1, for ideal relaxor ferroelectrics γ = 2, whereas in case of diffuse type of phase transition 1 ≤ γ ≤ 2. We have fitted the temperature dependent dielectric permittivity above the phase transition temperature by plotting the graph between *ln* $(\frac{1}{\varepsilon }-\frac{1}{{\varepsilon }_{max}})$ vs. ln (T − T_m_). By fitting the data the value of slope γ is found to be 1.6 for measurements at 10 kHz. This exponent suggests that the ferroelectric to paraelectric phase transition is diffuse in nature^43^. The ferroelectric transition temperature is found to shift towards higher temperature compared to the ferroelectric transition of pure PFN thin film (~ 400 K at 1 kHz) which might be due to the development of the strain at the interfaces of the heterostructure and strong mechanical coupling of ferroelectric phase with the ferromagnetic phase^20,21,40,44^. The increasing of ferroelectric T_c_ towards higher temperature with increasing concentration of NZFO in bulk multiferroic composite has already been reported^15,17^. As in composite structures, ferroelectric and magnetic order parameters are coupled, so both ferroelectric and magnetic properties are affected by the increase/decrease of ferroelectric and magnetic material content. This type of behavior, i.e., the increase of ferroelectric T_c_ with increase of magnetic content has also been theoretically predicted for multiferroic composite thin films^45^. For these heterostructures, the increase of ferroelectric Tc shifts towards higher temperature compared to pure PFN may be explained due to the effective misfit strain and ME coupling. In ferroelectric materials, the quadratic coupling between polarization and strain are particularly strong. This strong polarization-strain coupling is responsible for the changes in the ferroelectric T_c_. Due to the strong coupling between strain and ferroelectricity, T_c_ shifts of hundreds of degrees are theoretically predicted and have been experimentally observed in epitaxial strained ferroelectric thin films^46,47^. It is also reported that the development of strain, deforms the crystal in the perpendicular direction which provides space for ionic displacement of B site atoms, hence the T_c_ is expected to be modified^48^. In composite structures, the effective misfit strain on ferroelectric material is mechanically transmitted to the magnetic materials through the magnetostrictive effect inducing a change in the magnetic order parameters and T_c_^45^. Another interesting observation is that another anomaly at ~645 K at 100 kHz begins to develop and becomes prominent as the frequency increases; this is near the magnetic phase transition of the heterostructure. While this does not confirm the existence of magnetodielectric coupling, the presence of anomalies in both magnetic and dielectric susceptibilities at a similar temperature is intriguing. Interestingly, we also observed another hump near 400 K in the dielectric spectra, which may be due to onset of the ferroelectric phase transition. The inset of Fig. 4(a) shows the variation of loss tangent with temperature at different frequencies. The value of loss tangent is nearly constant up to 450 K and after that it increases rapidly with increase in temperature. The increase of dielectric loss is more prominent in low frequency regions as compared to high frequency regions at high temperature. It is well known that at high temperature thermally activated mobile charge carriers significantly influenced the ac conductivity and hence also the tangent loss and imaginary part of dielectric constant. The mobile charge carriers lag the high probe frequency (>1 kHz) hence negligible effect on the dielectric response. The room temperature loss tangent is found to be very low which makes this structure as suitable candidate for practical device.

To check the global nature of ferroelectricity and spontaneous polarization of these nanostructures, polarization versus electric field (*P-E*) measurements have been carried out at 10 kHz at room temperature as shown in Fig. 4(b). Well-saturated slim hysteresis has been observed with a saturation polarization (P_s_) and remanent polarization (P_r_) of 38 and 12 (+/−0.5) µC/cm^2^ respectively. The coercive field (H_c_) of this nanostructure was found to be 0.15 MV/cm. The saturated P-E loops confirm the intrinsic and integral ferroelectric nature of the trilayer films above room temperature.

### Magnetic Properties

**Figure 5:**
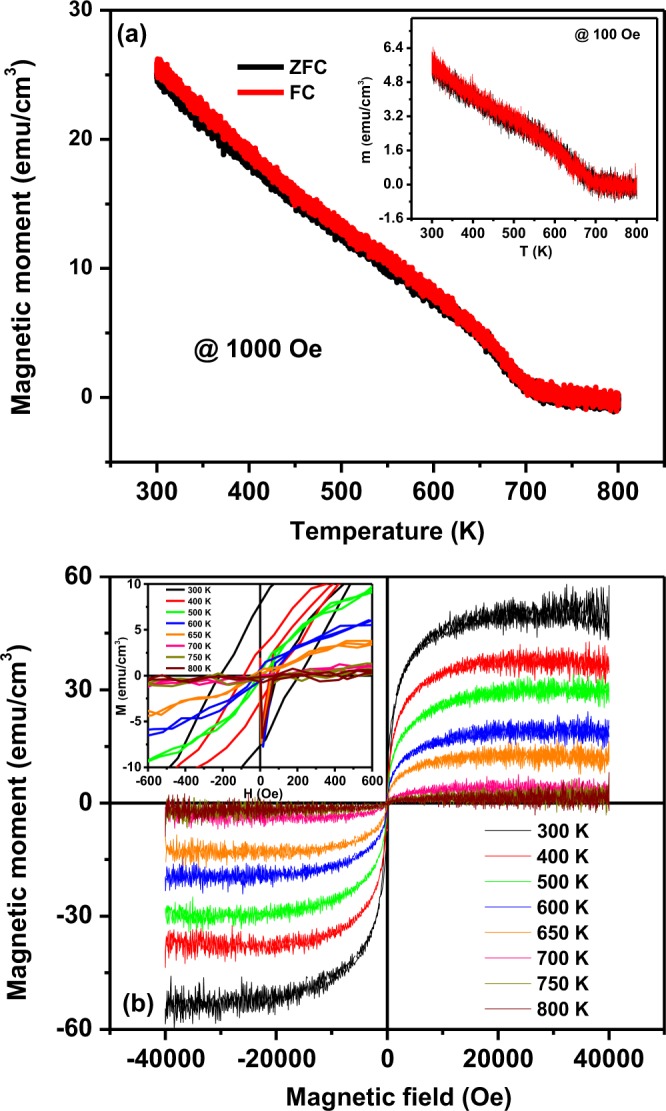
(**a**) Temperature dependence of magnetization measured with zero field cooling (ZFC) and field cooling (FC) with applied field of 1000 Oe (inset:100 Oe) (**b**) M–H hysteresis loops at different temperature of PFN/NZFO/PFN heterostructures.

In order to justify the existence of magnetic ordering above room temperature of PFN/NZFO/PFN heterostructures, dc-magnetization measurements have been carried out as a function of temperature M(T) and magnetic field M(H) as shown in Fig. 5(a,b), respectively. Both the M (H) and M (T) measurements carried out in the in-plane (magnetic field parallel to the thin film surface) configuration with external magnetic field of 100 Oe and 1000 Oe in a wide temperature range of 300–800 K as shown in Fig. 5(a). The M(T) measurements are recorded at two different modes: zero-field-cooled (ZFC) and field-cooled (FC). This heterostructure shows a faint bifurcation or hysteresis in the investigated temperature range in both 100 Oe (inset of Fig. 5(a)) and 1000 Oe which is characteristics of slightly frustrated ferromagnetic systems. It is observed from Fig. 5(a) that the magnetization decreases slowly with increase of temperature and vanishes above ~700 K. The ferro-/ferrimagnetic – paramagnetic transition temperature (T_c_) is found to be 650 (+/−10) K with a broad transition until 700 K which reveals the existence of short range spin interactions, which results in spin frustration and phase separation. In the existing literature, the reported antiferromagnetic (AFM) - paramagnetic (PM) phase transition of PFN single crystal and bulk ceramics is 140 to 155 K whereas for thin film it is 170–200 K^17,27,28,30,44^. The existence of weak ferromagnetism until 400 K has also been reported^49^. The magnetic Curie temperature of Ni_0.65_Zn_0.35_Fe_2_O_4_ ceramic is ~663 K^33,50^. The M(H) and M(T) measurements of both parent compounds were also carried out to get more insight to the magnetic properties. PFN thin films grown on LNO/LSAT exhibit weak ferromagnetic like behavior up to 400 K whereas the magnetic phase transition of NZFO thin film is found ~713 K. The saturation magnetization (Ms) of pure PFN and NZFO thin films of 140 nm were found to be 15 and 290 emu/cm^3^ at RT^51^. The fact that the magnetic transition temperature of the heterostructures is higher than that of PFN and NZFO might be due to the effective misfit strain experienced by each layer of the heterostructure and strong coupling of electrical and magnetic order parameters. Figure 5(b) depicts the magnetic hysteresis M(H) behavior of the hetero-structure up to ±40 kOe at different temperatures. The saturated magnetization is observed above ±10 kOe down to 300 K, which is a typical signature of ferro-/ferrimagnetic behavior. The square shape becomes more prominent with increase in temperature. The initial magnetization rapidly increases in low field regime (0–10 kOe) however, at high fields (>10 kOe) the magnetization growth weakens and saturates completely. The maximum saturation magnetization (M_S_) of the heterostructure at 300 K is found to be around 48(+/−1) emu/cm^3^ and gradually decreases with increase of temperature. The magnetization does not completely disappear above the phase transition up to 800 K confirming the presence of a ferro- (ferri) magnetic phase with paramagnetic relaxor background. The monotonic decrease in the magnitude of remnant magnetization (M_r_) and coercive field (H_c_) were also seen with increase of temperature. The enlarged M-H loops at low field are depicted in the inset of Fig. 5(b). The observed high saturation magnetization and low coercive field confirm the soft magnetic nature of the heterostructure, these features may be useful for designing of spintronic devices to switch the magnetic ordering with small external magnetic field. Hence it can be concluded that the trilayer heterostructures exhibit ferromagnetic-like behavior well above the room temperature.

### Magnetoelectric Coupling Properties

**Figure 6:**
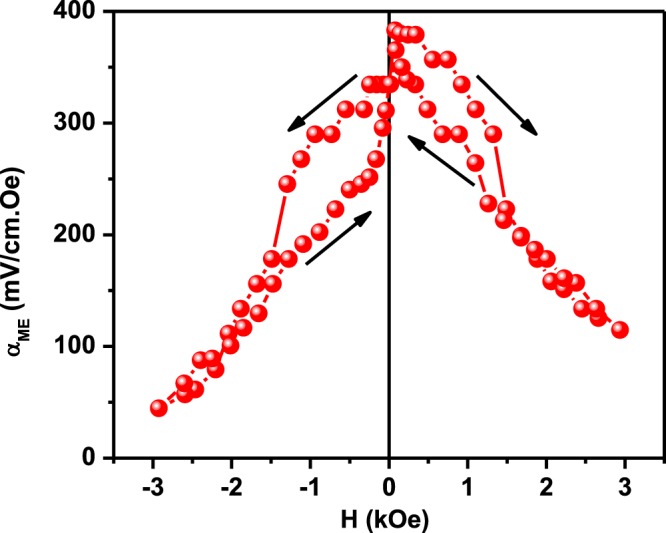
Magnetoelectric voltage coefficient as a function of the bias magnetic field in transverse mode for PFN/NZFO/PFN heterostructures at room temperature.

In order to prove the coupling between electrical and magnetic order parameters, the magnetoelectric coupling coefficient (α_ME_) of the trilayer heterostructure was measured by dynamic method at room temperature and shown in Fig. 6. For dynamic ME characterization, an ac field of *δH* = 1 Oe at 1 kHz and a bias magnetic field *H* were applied and the change in voltage δV across the trilayer was measured. The ME coupling coefficient was calculated using the empirical formula α_ME_ = *δE*/*δH* = *δV*/(*t* × *δH)*, where *δV* is the measured output voltage, *δH* is the applied AC magnetic field and *t* is the total thickness of the heterostructure. The highest ME coupling coefficient is found to be ~ 383 mV/cm.Oe at a bias field of +80 Oe followed by a sharp decrease with increasing of magnetic field. Similar type of behaviors were also observed in single phase multiferroic ceramics, epitaxial thin films and composite structures^52–56^. From the figure it is observed that α_ME_ value does not decrease to zero value with increasing H. The ME voltage coefficients (α_ME_) is directly proportional to the product of the piezomagnetic coefficient q = d*λ*/dH (where *λ* is the magnetostriction) and the piezoelectric coefficient d. As the ME voltage arises due to magnetic-mechanical-electrical interactions, α_ME_ is proportional to q × d, its H_dc_ dependence is expected to follow the slope of *λ* vs H_dc_ of the magnetic material^8,9,24^. The magnetostriction of NZFO is expected to be stronger at lower H in these heterostructures and decreases with increasing H. A similar type of magnetic field dependence of magnetostriction behavior of nickel zinc ferrite has been reported^55^. In our case the increase or decrease of α_ME_ with H is not similar with the application of positive and negative magnetic field. This asymmetry in the ME coefficient might be due to the existence of magnetic anisotropy in this heterostructure and clamping of bottom ferroelectric layer with electrode coated substrate. It can also be from asymmetry in the piezoresponse, which is also noted in Fig. 3(a), although there are multiple possible origins for this offset. Regardless, our PFN/NZFO/PFN heterostructures exhibit a ME coupling coefficient (α_E,31_) ~ 383 mV/cm.Oe which is significantly higher than the superlattices of Ba_0.7_Sr_0.3_TiO_3_/La_0.7_Sr_0.3_MnO_3_ (α_E,31_ ~ 250 mV/cm.Oe), Ni/PZT/Ni trilayer heterostructure (α_E,31_ = 270 mV/cm.Oe) CoFe_2_O_4_/BaTiO_3_ bilayer (α_E,31_ ~ 104 mV/cm.Oe) NiFe_2_O_4_/BaTiO_3_ multilayer heterostructures (α_E,31_ ~ 90 mV/cm.Oe), Pb (Zr_0.4_Ti_0.6_)O_3_–Ni_0.8_Zn_0.2_Fe_2_O_4_ multilayered thin films (α_E,33_ ~ 15 mV/cm.Oe), (1 − x) BiFeO_3_- x BaTiO_3_ composite thin films(α_E,31_ ~ 124 mV/cm.Oe), BiFeO_3_/BaTiO_3_ bilayer ((α_E,33_ ~ 61 mV/cm.Oe)^23,56–61^ We observed strong ME coupling as these heterostructures exhibit strong polarization and saturation magnetization at room temperature and the perfect strain transmission between the ferroelectric and magnetic layers due to epitaxial growth^2,7–9,20,24^. The mechanism responsible for the strong magnetoelectric effect in this case might be indirectly via elastic contribution rather than a direct coupling between the magnetic and electric order parameters of both ferroelectric and magnetic layers. By the application of magnetic field, the magnetic phase shows a magnetostriction effect and produces strain in the system. That strain induces a piezoelectric effect in the ferroelectric phase, which changes the electrical order parameter and hence output ME signal^2,7,8,55–60^. In the layered thin film composite structures, giant magneto-electric effects can also be induced due to large interfacial strain across two phases, change in oxygen stoichiometry, electronic correlations, interface bonding, exchange bias, etc. The electronic origin of the ME coupling in the nanostructure may arise from a change in the valence states of magnetic ions induced by electrostatic charge modulation. The large ME coupling effect found in these artificial heterostructures might also be due to interfacial magnetic reconstruction driven by charge accumulation^7–9,55–60,62^. The exact mechanisms behind the observed magnetoelectric behaviour requires substantial first principles and phenomenological investigations, which are currently underway. The value of such reasonably high ME coupling coefficient observed at room temperature paves the way for the design and development of new spintronic devices. These observations suggest that epitaxial growth of alternative ferromagnetic and ferroelectric layers offers a route towards achieving strong ME coupling. The thickness of the FM may be kept to below the thickness of the FE layer, to minimize leakage currents and dielectric loss. By controlling the strain state via magnetostriction, through changes in anisotropy and magnetic moment the strong coupling to the polarization in the ferroelectric layers can be achieved. Multilayer structure of strongly piezoelectric materials such as lead zirconate titanate (PZT) and other relaxor ferroelectrics with ferromagnetic materials which exhibits higher magnetostriction coefficient than nickel zinc ferrite can be envisioned, although challenges would be in transferring the strain across the interfaces. Such ME multilayer heterostructures and their combination of properties might hold the future for the ultimate multifunctional and spintronics devices.

## Conclusions

Three layer epitaxial FE/FM/FE sandwich nanostructures are grown on LSAT single crystal substrate. The global single-crystalline nature of the hetero-structure is observed with large strain across the interfaces. The existence of nanoscale ferroelectricity, sharp polarization switching at low coercive field and tip induced polarization switching has been confirmed by band excitation PFM studies. The distribution of the ferroelectric loop area is almost uniform in a wide area, suggesting that spatial variability of ferroelectric switching behavior is low, and film growth is of high quality. The ferroelectric and magnetic phase transitions have been probed above room temperature. The nanostructures show saturated large polarization, soft magnetization, low loss tangent and large ME coupling at room temperature which may be useful for nanoscale multifunctional and spintronics device applications.

## Methods

The ceramic targets of PFN and NZFO were prepared by a conventional high temperature solid state reaction route. The synthesis conditions and structural properties of each compound are reported elsewhere^15,17,31^. PFN/NZFO/PFN heterostructures were grown on LaNiO_3_ (LNO) bottom electrode deposited on commercially available (LaAlO_3_)_0.3_ (Sr_2_AlTaO_6_)_0.7_ (LSAT) (100) substrates utilizing optimized pulsed laser deposition (PLD) with an excimer laser (KrF, 248 nm). Initially 90 nm of LNO bottom electrode was deposited at 700 °C in an oxygen ambient of 200 mTorr, followed by annealing in same oxygen ambient of 300 Torr for 30 minutes at same temperature and then slowly cooled down to room temperature. PFN and NZFO layers were deposited at a fixed temperature of 600 °C under an oxygen pressure of 20 mTorr and 150 mTorr, respectively with a laser energy density ~1.5 J/cm^2^. Later, the PFN/NZFO/PFN heterosrtructure was annealed in oxygen atmosphere of 300 Torr for 30 minutes at 700 °C and later cooled down to room temperature slowly. The thickness of both PFN layers and the middle NZFO layer were found to be around 70 nm and 20 nm, respectively.

The epitaxial quality of PFN/NZFO/PFN heterostructures was confirmed by high resolution XRD using CuK_α_ radiation with wavelength of λ = 1.5405 Å operated at a scan rate of 1 °/min over the angular range (2θ) of 20–80 at room temperature. The thicknesses of the films were measured utilizing XP-200 profilometer along with TEM. For TEM imaging, the lamellae were cut from the PFN/NZFO/PFN heterostructure, and were lifted using a fine glass needle and micromanipulator under an optical microscope, and transferred onto a carbon coated 3 mm copper-mesh grid. Imaging of heterostructure was then performed using a scanning transmission electron microscopy (STEM-Philips CM 200 (FEG)) on a 200 kV field-emission transmission electron microscope, using a high-angle annular dark-field detector. Specimen imaging was performed with the electron beam perpendicular to the lamellar face. The local chemistry at the PFN and NZFO layers was investigated using STEM-EDS in a Philips CM 200 (FEG) operated at 200 kV. The PFM spectroscopy experiments were performed by moving the tip across a pre-defined coordinate (x,y) grid on the sample and perturbing it with a DC bias waveform, and then measuring the strain (piezoresponse) as a function of frequency via Band Excitation (BE)^41,42,63^. BE technique involves the generation of a band of AC frequencies around the contact resonance of the cantilever and measuring the electromechanical response over time with subsequent Fourier transformation back to the frequency domain to yield the frequency-dependent response. This measurement is repeated for several time steps after each DC bias pulse; thus, at each (x,y) position, local spectroscopic information is acquired as a function of perturbation voltage (V) and excitation frequency (f), characterizing the system’s mechanical response (vertical deflection) R = R(x, y, V, f). The response R at each (x, y, V) step is fit to a simple harmonic oscillator (SHO) function yielding the amplitude (A), phase(φ), quality factor (Q), and resonant frequency (ω) associated with the response. The thin film was glued onto a sample plate with silver epoxy, which acted as the bottom electrode and was grounded for the PFM experiments. The PFM experiments were performed at room temperature with moderately stiff Budget Sensors ElectriMulti75-G cantilevers (k ∼ 1 N/m) and a free resonance (in air) of ∼75 kHz, on a Multimode (Veeco) AFM equipped with a Nanonis controller. National Instruments DAQ cards were employed for signal generation and acquisition for the band-excitation experiments, which were performed using scripts written in Labview v11 and Matlab version 2011. All analyses were carried out utilizing Matlab version 2011. Temperature dependent magnetic properties of the heterostructure were carried out using a PPMS DynaCool (Quantum design) in a wide temperature range of 300–800 K. The room temperature ME measurements were performed using a magnet with a varying field of up to ±0.5 T with a lock-in amplifier and reference ac magnetic field using Helmholtz coil. Pt top electrodes of area ~10^−4^ cm^2^ and thickness ~60 nm were made by dc sputtering utilizing a metal shadow mask for electrical characterization. Dielectric parameters, i.e., capacitance, loss tangent, impedance, and phase angles, were measured from 80 to 700 K in a wide frequency range of 100 Hz to 1 MHz using an impedance analyzer HP4294A with MMR Technologies K-20 programmable temperature controller with fixed ac voltage amplitude of 0.1 V. Ferroelectric (P−E) loops were measured using Precision Multiferroic and Ferroelectric Test System (Radiant Technologies Inc., Albuquerque, NM, USA) at room temperature.
